# Machine learning-based models to predict 90-day non-excellent functional outcomes among patients with mild acute ischemic stroke after intravenous thrombolysis

**DOI:** 10.3389/fneur.2026.1960403

**Published:** 2026-10-19

**Authors:** Yichen Wang, Xinyu Qiu, Yi Wang, Lijun Wang

**Affiliations:** 1 Tianjin Medical University, Tianjin, China; 2 Tianjin University, Tianjin, China; 3 Tianjin Fourth Central Hospital Affiliated to Tianjin Medical University, Tianjin, China

**Keywords:** explainable artificial intelligence, functional outcome, intravenous thrombolysis, machine learning, mild acute ischemic stroke

## Abstract

**Background:**

Despite intravenous thrombolysis, a substantial proportion of patients with mild acute ischemic stroke fail to achieve an excellent functional outcome. Prediction models specifically developed for these patients remain limited. We aimed to construct and evaluate a machine learning model for identifying 90-day non-excellent functional outcomes.

**Methods:**

We retrospectively collected data from patients with mild acute ischemic stroke treated with intravenous thrombolysis at Tianjin Fourth Central Hospital between January 2024 and June 2025. Demographic, clinical, laboratory, and neuroimaging variables were extracted from electronic medical records. LASSO regression was applied for feature selection, followed by the development of seven machine learning models. Model performance was assessed using discrimination, calibration, clinical utility, and classification metrics. We conducted SHAP analysis to elucidate the relative importance and direction of association of individual predictors with model predictions.

**Results:**

A total of 507 patients with mild acute ischemic stroke undergoing intravenous thrombolysis were included, of whom 136 (26.8%) experienced a non-excellent functional outcome at 90 days. LASSO regression identified six predictors, including age, baseline National Institutes of Health Stroke Scale score, platelet count, monocyte count, blood glucose level, and neutrophil-to-lymphocyte ratio. In the validation cohort, XGBoost achieved the highest numerical AUC of 0.753 (95% CI, 0.660–0.840) and demonstrated adequate calibration and clinical usefulness.

**Conclusion:**

A machine learning model was developed and internally validated to predict 90-day non-excellent functional outcomes in patients with mild acute ischemic stroke after intravenous thrombolysis. The XGBoost model showed promising predictive performance and potential utility for early risk stratification.

## Introduction

Globally, acute ischemic stroke (AIS) continues to represent a leading contributor to both fatal outcomes and sustained functional impairment, comprising roughly seven to eight out of every ten stroke incidents. The GBD 2021 study estimated that stroke contributed to roughly 11.9 million new cases per year, ranking as the world’s second most common cause of mortality and the third most significant source of disability-adjusted life years (DALYs) (1, 2). Intravenous thrombolysis (IVT) is currently one of the cornerstone reperfusion therapies for AIS and has been proven to improve functional outcomes (3). Mild neurological impairment, defined as a National Institutes of Health Stroke Scale (NIHSS) score of 5 or lower, is observed in roughly 50% of patients with AIS (4). Despite receiving timely IVT, an estimated 20 to 35% of patients with minor AIS fall short of excellent functional status (modified Rankin Scale [mRS] score of 0–1) at 90 days (5). Even among patients with mild stroke, failure to achieve an excellent functional outcome may adversely affect functional independence and health-related quality of life, highlighting the need for early identification of patients at increased risk of poor recovery (4). Functional outcomes following mild AIS are determined by the complex interplay of multiple clinical factors and exhibit considerable interindividual variability (6). At present, prognostic assessment largely depends on neurological deficit severity and clinicians’ judgment, while validated risk stratification tools specifically developed for patients with mild AIS undergoing IVT remain limited, hindering individualized prognostic evaluation and early clinical decision-making.

In recent years, machine learning (ML) has been increasingly applied to prognostic prediction in stroke (7, 8). Unlike traditional statistical modeling, machine learning excels at navigating high-dimensional data spaces and uncovering sophisticated nonlinear associations among predictors, making it a compelling option for personalized prognostic model development (9). The increasing use of interpretable artificial intelligence methods, including Shapley Additive Explanations (SHAP), has made ML models more transparent by revealing the contribution of individual predictors to prediction outcomes, thereby improving their interpretability and facilitating clinical implementation (10).

Several studies have applied ML algorithms to predict functional outcomes after intravenous thrombolysis in patients with AIS and have reported encouraging predictive performance. However, these studies have several important limitations. First, most enrolled heterogeneous populations comprising patients with varying stroke severity, limiting the generalizability of their findings to patients with mild AIS (11). Second, many existing models incorporated in-hospital variables or post-treatment laboratory parameters (12, 13). Although these variables may improve predictive performance, they substantially limit the utility of such models for pre-treatment risk stratification before intravenous thrombolysis. Furthermore, most previous studies defined unfavorable outcome (mRS ≥ 3) or early neurological deterioration (END) as the primary endpoint (14). However, for patients with mild AIS, even those with a 90-day mRS score of 2 may experience impaired functional independence, reduced work capacity, and ongoing rehabilitation needs. Therefore, failure to achieve an excellent functional outcome (90-day mRS score ≥2) may represent a more clinically meaningful endpoint for evaluating functional recovery and long-term prognosis in this patient population.

In this study, we enrolled patients with mild AIS who underwent IVT and collected pre-thrombolysis clinical characteristics and routine laboratory parameters. A machine learning–based prediction model was developed and validated to estimate the risk of a non-excellent functional outcome at 90 days. Model performance was assessed comprehensively by evaluating its discrimination, calibration, clinical usefulness, and interpretability. This study sought to establish a practical tool for risk stratification in patients with mild AIS undergoing intravenous thrombolysis, thereby facilitating early prognostic assessment and individualized clinical management.

## Methods

### Data source

This single-center retrospective study included consecutive patients diagnosed with AIS who received IVT at Tianjin Fourth Central Hospital between January 2024 and June 2025. Clinical information was retrieved from the hospital’s electronic medical records. Eligible participants were selected according to the following criteria. The inclusion criteria were as follows: (1) be at least 18 years of age; (2) a diagnosis of AIS according to the Chinese Guidelines for the Diagnosis and Treatment of Acute Ischemic Stroke (15) and verified by either computed tomography (CT) or magnetic resonance imaging (MRI); (3) receipt of IVT with alteplase or tenecteplase after symptom onset; and (4) a baseline NIHSS score ≤5 on admission (16). Patients were excluded if they met any of the following criteria: (1) receipt of bridging endovascular therapy or mechanical thrombectomy; (2) pre-existing significant neurological disability, defined as a pre-stroke mRS score >1; (3) concomitant malignant tumor, severe infection, autoimmune disease, hematological disorder, or severe hepatic or renal dysfunction; and (4) missing key clinical or laboratory data. The study protocol was approved by the Research Ethics Committee of Tianjin Fourth Central Hospital (Approval No. SZXLL-2026-KY001) and was performed in accordance with the principles of the revised Declaration of Helsinki.

### Data collection

Clinical information was retrospectively retrieved from the electronic medical record database of Tianjin Fourth Central Hospital. Data collected for the analysis covered demographic information (age and sex), comorbidities (hypertension, diabetes mellitus, coronary heart disease, atrial fibrillation, antiplatelet history, smoking status, and alcohol consumption), clinical characteristics, laboratory parameters, and neuroimaging findings. Clinical characteristics comprised onset-to-treatment time (OTT), admission NIHSS score, systolic blood pressure (SBP), and diastolic blood pressure (DBP). Laboratory parameters included complete blood count, blood glucose, blood biochemical parameters, lipid profile, and electrolytes. The neutrophil-to-lymphocyte ratio (NLR), an inflammation-related biomarker, was calculated according to previously published methods. Neuroimaging data, including admission cranial CT, MRI, and computed tomography angiography (CTA), were reviewed to determine the responsible vascular territory and the Trial of Org 10,172 in Acute Stroke Treatment (TOAST) etiological subtype. The responsible vascular territory was determined by integrating imaging findings with clinical signs and symptoms and was independently assessed by two experienced stroke neurologists. Any discrepancies were resolved by consensus. Based on imaging findings, the responsible vascular territory was classified as either anterior circulation or posterior circulation.

### Clinical outcome

The primary outcome was a non-excellent functional outcome, defined as a modified Rankin Scale (mRS) score of 2–6 at 90 days after intravenous thrombolysis. Functional outcomes were assessed at 90 days after stroke onset using the mRS by neurologists who had received standardized training. The assessment was performed either during outpatient follow-up visits or through structured telephone interviews. The mRS assessors were independent of the model development process and were unaware of the model predictions. Participants were classified as having an excellent functional outcome (mRS score 0–1) or a non-excellent functional outcome (mRS score 2–6) (17).

### Sample size

As this was a single-center retrospective study, no formal sample size calculation was performed before data collection. Instead, all consecutive patients who met the eligibility criteria during the predefined study period were included. After LASSO feature selection, six predictors were retained, and the number of outcome events exceeded 10 per predictor in the final model (18).

### Data preprocessing

Patients with missing key clinical or laboratory data or unavailable 90-day follow-up data were excluded from the analysis, and no data imputation was performed. Using the createDataPartition function from the caret package in R, the dataset was divided into a training cohort (70%) and an internal validation cohort (30%) using stratified random sampling according to the outcome variable. A predefined random seed (seed = 123) was set before data partitioning to ensure reproducibility. All feature selection, model training, cross-validation, and hyperparameter optimization procedures were performed exclusively within the training cohort. After model development, the internal validation cohort was used to evaluate the predictive performance of the candidate models developed from the training cohort. Hereafter, the internal validation cohort is referred to as the validation cohort for brevity.

### Feature selection

In the training cohort, the Least Absolute Shrinkage and Selection Operator (LASSO) logistic regression was performed for feature selection (19). Continuous predictors were normalized using min–max scaling before LASSO analysis. The optimal penalty parameter (λ) was determined using 10-fold cross-validation, and the λ1se criterion was used to select the penalty parameter for feature selection. Predictors with non-zero coefficients at λ1se were retained for subsequent machine-learning model development. Six predictors were ultimately selected.

### Machine learning model development

The training cohort was used to develop seven prediction models based on different machine learning algorithms: Logistic Regression (LR), Decision Tree (DT), Random Forest (RF), eXtreme Gradient Boosting (XGBoost), Light Gradient Boosting Machine (LightGBM), Support Vector Machine (SVM), and Artificial Neural Network (ANN). LR was implemented using the standard Logit model in the statsmodels package and fitted by maximum likelihood estimation. As the conventional logistic regression model used in this study did not involve model-complexity hyperparameters requiring optimization, no additional grid-search tuning was performed. For the remaining six machine-learning models, hyperparameter optimization was performed using grid search combined with stratified five-fold cross-validation. Specifically, the training cohort was partitioned into five folds; for each candidate hyperparameter combination, four folds were used for model fitting and the remaining fold for evaluation, with this process repeated across all five folds. Five-fold cross-validation was selected to balance the stability of performance estimation with computational efficiency when evaluating multiple hyperparameter combinations across several algorithms. Hyperparameter search spaces were predefined according to the characteristics of each algorithm. The mean area under the receiver operating characteristic curve (AUC) across the five validation folds was used as the optimization criterion, and the hyperparameter combination yielding the highest mean AUC was selected as the optimal configuration. Model development and evaluation were conducted using the original outcome distribution without resampling. After hyperparameter optimization, the six tuned models were refitted on the entire training cohort using their respective optimal hyperparameter configurations, whereas the LR model was fitted directly on the entire training cohort using the prespecified Logit specification. All seven finalized models were subsequently evaluated on the internal validation cohort.

The models were implemented using the scikit-learn, XGBoost, LightGBM, and statsmodels packages. A random seed of 123 was specified where applicable to improve reproducibility. The ANN was implemented using a multilayer perceptron classifier with two hidden layers of 100 neurons each and a logistic activation function, with a maximum of 500 iterations. Detailed hyperparameter search spaces and optimal settings for all models are provided in Supplementary Table 1.

Model discrimination was evaluated using receiver operating characteristic (ROC) curves and AUC, with 95% confidence intervals (95% CIs) calculated for each model. The 95% confidence intervals for the AUCs were estimated using 1,000 bootstrap resamples with replacement. In addition, accuracy, sensitivity, specificity and the F1-score were calculated to comprehensively assess classification performance. A probability threshold of 0.30 was used to convert predicted probabilities into binary classifications for the calculation of accuracy, sensitivity, specificity, F1 score, PPV, and NPV. Model calibration was assessed using calibration curves with 10 probability bins and the Brier score. Clinical utility was evaluated using decision curve analysis (DCA) by comparing the net benefit of each model with the treat-all and treat-none strategies across threshold probabilities ranging from 0.01 to 0.99.

### SHAP interpretability analysis

Following comparative evaluation of the finalized candidate models, the model prioritized for subsequent interpretability analysis was further analyzed using SHapley Additive exPlanations (SHAP) (20). SHAP analysis was implemented using the SHAP package in Python with a model-agnostic KernelExplainer. The internal validation cohort was used for model interpretation. For the binary classification task, SHAP values corresponding to the predicted probability of a 90-day non-excellent functional outcome were analyzed. Global model interpretability was assessed using SHAP feature importance bar plots and beeswarm plots to characterize the relative contribution of each predictor to the model output. Dependence plots were generated for all predictors included in the interpreted model to characterize the relationships between predictor values and their corresponding SHAP values.

### Statistical analysis

We conducted all statistical analyses in R (version 4.5.1) and Python (version 3.10.4). The principal R packages included caret, glmnet, tableone, and pROC, whereas Python-based modeling and interpretability analyses were performed using scikit-learn, XGBoost, LightGBM, statsmodels, and SHAP packages. Baseline characteristics were compared between patients with excellent functional outcomes (mRS 0–1) and those with non-excellent functional outcomes (mRS 2–6) to characterize differences in baseline clinical characteristics between the two outcome groups. Standardized mean differences (SMDs) were calculated to quantify the magnitude of baseline differences between patients with excellent and non-excellent functional outcomes. The Shapiro–Wilk test was employed to examine the normality of continuous data. Continuous variables with a normal distribution were expressed as mean ± standard deviation (SD), and comparisons between groups were performed with the independent-samples *t* test. Continuous variables that did not follow a normal distribution were expressed as the median with interquartile range (IQR), and group comparisons were performed using the Mann–Whitney U test. Categorical variables were presented as counts and percentages and compared using the chi-square test when expected cell frequencies were adequate for the chi-square approximation or Fisher’s exact test when they were insufficient. All statistical tests were two-sided, and a *P* value <0.05 was considered statistically significant.

## Results

### Patient characteristics

From January 2024 to June 2025, a total of 531 patients with mild acute ischemic stroke who underwent intravenous thrombolysis were initially screened. Of these, 24 patients were excluded: 11 because of bridging endovascular therapy, 6 because of pre-existing significant neurological disability, and 7 because of missing data. Among the 7 patients excluded because of missing data, 3 had missing key clinical or laboratory data and 4 had unavailable 90-day follow-up data. Consequently, 507 patients were included in the final analysis (Figure 1). Among these patients, 498 received alteplase and 9 received tenecteplase. Table 1 presents the baseline characteristics of patients with mild acute ischemic stroke who underwent IVT, and the corresponding SMDs are provided in Supplementary Table 3. Among the 507 patients included, 136 (26.8%) had a non-excellent functional outcome (mRS score of 2–6) at 90 days. The median age of the overall cohort was 69.0 years (IQR, 62.0–74.0), and 311 patients (61.3%) were male. Compared with patients who achieved an excellent functional outcome, those with a non-excellent functional outcome were older and had higher baseline NIHSS scores, systolic and diastolic blood pressure, white blood cell counts, monocyte counts, neutrophil counts, blood glucose levels, and neutrophil-to-lymphocyte ratios, whereas lymphocyte counts were lower (all *p* < 0.05). In addition, the prevalence of atrial fibrillation and the distribution of TOAST etiological subtypes differed between the two groups (both *p* < 0.05).

**Figure 1:**
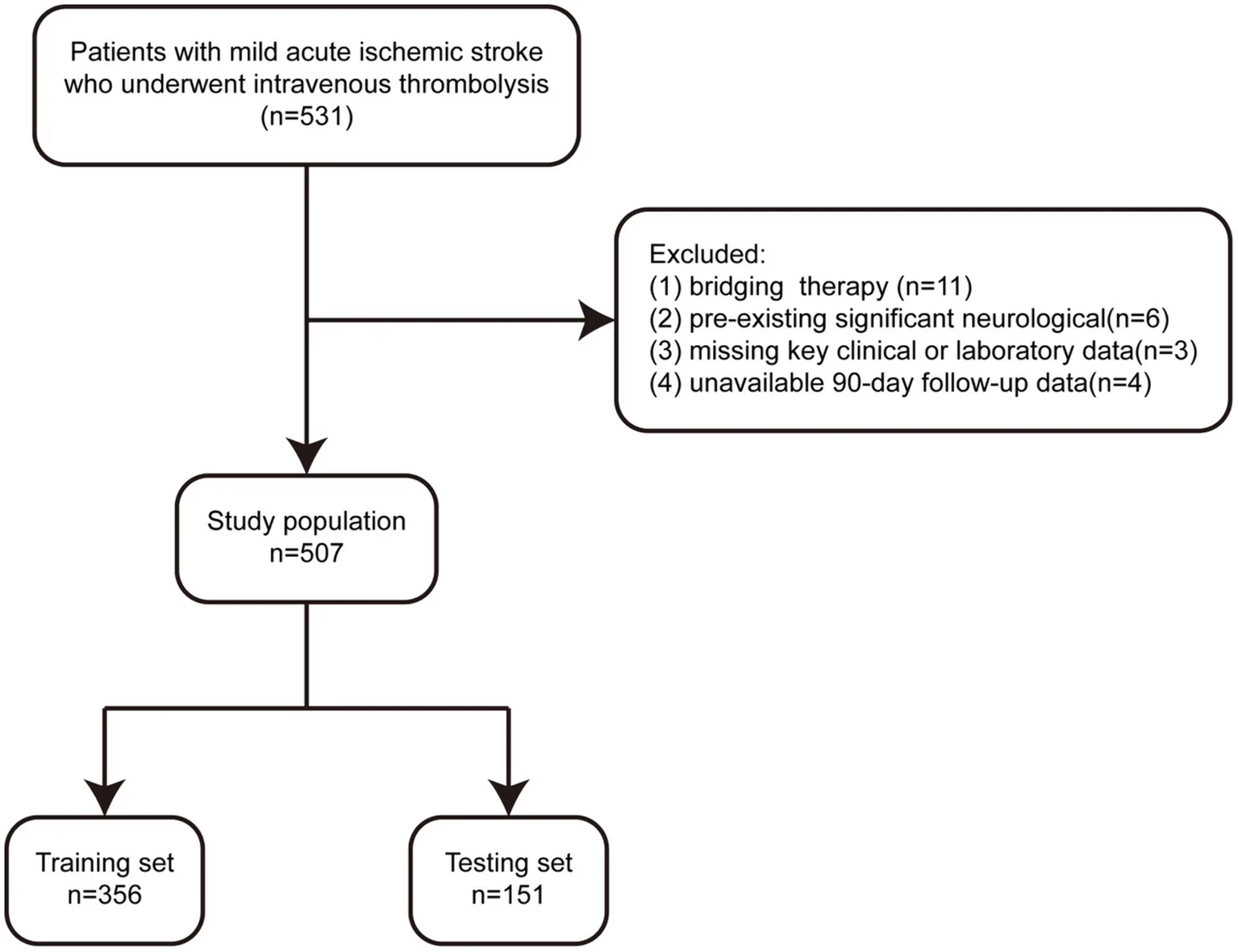
The flow chart of the participants selection.

**Table 1:** Baseline characteristics of patients with mild acute ischemic stroke undergoing intravenous thrombolysis.

Variables	Total (*n* = 507)	Excellent outcome (*n* = 371)	Non-excellent outcome (*n* = 136)	*p*
Male (%)	311 (61.34)	228 (61.46)	83 (61.03)	0.93
HT (%)	354 (69.82)	250 (67.39)	104 (76.47)	0.062
DM (%)	159 (31.36)	107 (28.84)	52 (38.24)	0.056
CHD (%)	225 (44.38)	157 (42.32)	68 (50.00)	0.149
AF (%)	48 (9.47)	26 (7.01)	22 (16.18)	0.003
Smoking (%)	199 (39.25)	146 (39.35)	53 (38.97)	0.938
Alcohol (%)	133 (26.23)	97 (26.15)	36 (26.47)	0.941
Antiplatelet (%)	480 (94.67)	352 (94.88)	128 (94.12)	0.909
Responsible artery (%)				0.627
Anterior circulation	317 (62.52)	231 (62.26)	86 (63.24)	
Posterior circulation	160 (31.56)	120 (32.35)	40 (29.41)	
AC + PC	30 (5.92)	20 (5.39)	10 (7.35)	
TOAST (%)				<0.001
LAA	259 (51.08)	182 (49.06)	77 (56.62)	
SAO	221 (43.59)	179 (48.25)	42 (30.88)	
CE	22 (4.34)	7 (1.89)	15 (11.03)	
SOE	5 (0.99)	3 (0.81)	2 (1.47)	
Age (median [IQR])	69.00 [62.00, 74.00]	68.00 [61.00, 73.00]	71.00 [66.00, 77.00]	<0.001
OTT (median [IQR])	120.00 [72.50, 180.00]	120.00 [60.00, 155.00]	120.00 [90.00, 180.00]	0.067
NIHSS (median [IQR])	3.00 [2.00, 4.00]	3.00 [2.00, 4.00]	4.00 [3.00, 5.00]	<0.001
SBP (median [IQR])	150.00 [136.00, 165.00]	150.00 [135.00, 164.00]	155.50 [143.00, 165.00]	0.003
DBP (median [IQR])	80.00 [75.00, 90.00]	80.00 [74.00, 90.00]	81.50 [78.75, 90.00]	0.032
WBC (median [IQR])	7.03 [5.70, 8.50]	6.80 [5.53, 8.16]	7.52 [6.35, 9.45]	<0.001
PLT (median [IQR])	205.00 [169.50, 243.00]	203.00 [167.50, 241.00]	209.00 [173.75, 256.25]	0.05
LYM (median [IQR])	1.60 [1.18, 2.14]	1.61 [1.24, 2.16]	1.52 [0.99, 2.04]	0.013
MO (median [IQR])	0.46 [0.36, 0.58]	0.44 [0.35, 0.57]	0.51 [0.37, 0.64]	0.001
NE (median [IQR])	4.45 [3.33, 5.86]	4.24 [3.23, 5.54]	5.13 [3.93, 7.08]	<0.001
ALB (median [IQR])	37.80 [36.05, 39.70]	37.90 [36.40, 39.60]	37.70 [35.40, 39.90]	0.35
TC (mean (SD))	4.84 (1.18)	4.85 (1.19)	4.80 (1.17)	0.659
TG (median [IQR])	1.38 [1.04, 1.89]	1.39 [1.06, 1.86]	1.35 [0.93, 2.08]	0.941
LDL (mean (SD))	2.73 (0.90)	2.74 (0.90)	2.71 (0.90)	0.706
HDL (median [IQR])	1.08 [0.90, 1.25]	1.08 [0.90, 1.25]	1.07 [0.89, 1.25]	0.653
Glucose (median [IQR])	5.61 [4.93, 7.19]	5.39 [4.82, 6.53]	6.66 [5.46, 8.52]	<0.001
Hb (median [IQR])	130.00 [103.00, 142.00]	130.00 [100.00, 141.00]	129.00 [108.75, 143.00]	0.462
ALT (median [IQR])	15.00 [12.00, 21.00]	16.00 [12.00, 21.00]	15.00 [11.00, 21.00]	0.364
AST (median [IQR])	18.00 [14.00, 22.00]	17.00 [14.00, 21.00]	18.40 [14.00, 22.00]	0.205
Ca (median [IQR])	2.26 [2.19, 2.33]	2.25 [2.19, 2.32]	2.26 [2.18, 2.35]	0.613
K (median [IQR])	3.90 [3.60, 4.20]	3.90 [3.60, 4.15]	3.90 [3.60, 4.20]	0.691
NLR (median [IQR])	2.69 [1.79, 4.17]	2.52 [1.71, 3.70]	3.31 [1.92, 6.24]	<0.001

Data are presented as median (interquartile range), mean (standard deviation), or number (percentage). HT, hypertension; DM, diabetes mellitus; CHD, coronary heart disease; AF, atrial fibrillation; AC, anterior circulation; PC, posterior circulation; LAA, large artery atherosclerosis; SAO, small artery occlusion; CE, cardioembolism; SOE, stroke of other established etiology; OTT, onset-to-treatment time; NIHSS, National Institutes of Health Stroke Scale; SBP, systolic blood pressure; DBP, diastolic blood pressure; WBC, white blood cell count; PLT, platelet count; LYM, lymphocyte count; MO, monocyte count; NE, neutrophil count; ALB, albumin; TC, total cholesterol; TG, triglycerides; LDL, low-density lipoprotein cholesterol; HDL, high-density lipoprotein cholesterol; Hb, hemoglobin; ALT, alanine aminotransferase; AST, aspartate aminotransferase; Ca, serum calcium; K, serum potassium; NLR, neutrophil-to-lymphocyte ratio.

### LASSO variable selection

All candidate variables were included in the LASSO regression analysis. Using the one-standard-error criterion, the optimal penalty parameter was identified at λ.1se = 0.0481301, resulting in the selection of six predictors: age, baseline NIHSS score, platelet count, monocyte count, blood glucose level, and neutrophil-to-lymphocyte ratio (Figures 2A,B).

**Figure 2:**
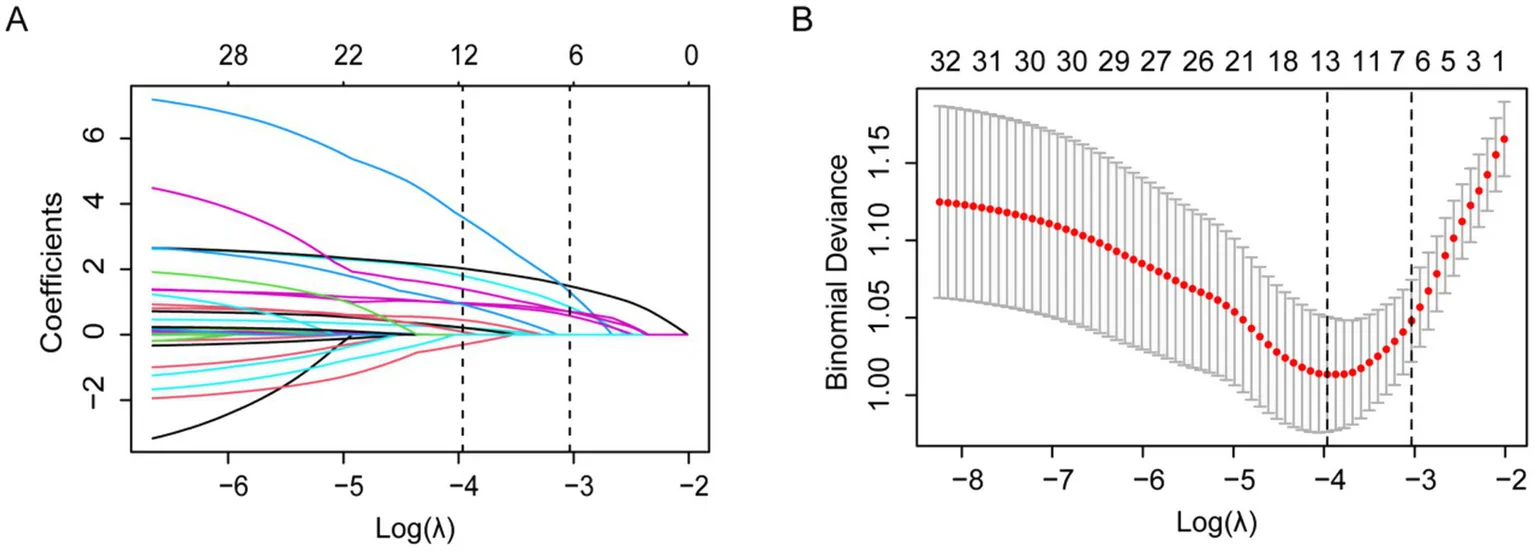
Feature selection by the LASSO regression model. **(A)** Coefficient path plot showing the trajectory of each candidate predictor’s regression coefficient across different λ values. Six predictors with non-zero coefficients at λ.1se were retained for subsequent model development: age, baseline NIHSS score, platelet count, monocyte count, blood glucose level, and neutrophil-to-lymphocyte ratio. **(B)** Ten-fold cross-validation error curve as a function of the penalty parameter λ. The dashed vertical lines indicate the λ value corresponding to the minimum mean cross-validated error (λ.min) and the optimal λ selected by the one-standard-error criterion (λ.1se = 0.0481301).

### Multimodel integrated analysis for classification

The six selected predictors were used to develop seven machine learning models for predicting non-excellent functional outcomes. The performance of the models is summarized in Table 2, with the corresponding 95% confidence intervals for the classification metrics provided in Supplementary Table 2. In the validation cohort, all models demonstrated predictive capability, although their performance varied. The decision tree model showed the lowest discriminative ability (AUC = 0.614), whereas the remaining models achieved AUC values above 0.73. The XGBoost model achieved an AUC of 0.753 (95% CI, 0.660–0.840), followed by the SVM, logistic regression, LightGBM, ANN, and random forest models, with AUCs of 0.750 (95% CI, 0.653–0.829), 0.747 (95% CI, 0.653–0.827), 0.746 (95% CI, 0.654–0.829), 0.745 (95% CI, 0.637–0.828), and 0.735 (95% CI, 0.639–0.824), respectively. Among the seven models, XGBoost yielded the highest AUCs in both the training and validation cohorts (Figures 3A,B). Calibration curves are presented in Figure 3C. The XGBoost model had a Brier score of 0.165 (95% CI, 0.127–0.207), which was comparable to those of the LR, RF, SVM, and ANN models, whereas the DT model had the highest Brier score (0.209), indicating relatively poorer calibration. Decision curve analysis showed that all models provided clinical net benefit across a range of threshold probabilities (Figure 3D). Overall, the differences in performance among the models were modest. Considering the overall performance of the finalized candidate models in the internal validation cohort, XGBoost showed the numerically highest AUC together with comparable calibration and clinical utility and was therefore selected for subsequent SHAP-based interpretation.

**Table 2:** The predictive performance of seven models.

Model	AUC	Accuracy	Sensitivity	Specificity	F1 Score	PPV	NPV
Logistic	0.747	0.715	0.550	0.775	0.506	0.468	0.827
Decision Tree	0.614	0.709	0.350	0.838	0.389	0.438	0.782
Random Forest	0.735	0.722	0.525	0.793	0.500	0.477	0.822
XGBoost	0.753	0.748	0.525	0.829	0.525	0.525	0.829
LightGBM	0.746	0.709	0.475	0.793	0.463	0.452	0.807
SVM	0.750	0.748	0.550	0.820	0.537	0.524	0.835
ANN	0.745	0.762	0.500	0.856	0.526	0.556	0.826

AUC, area under the receiver operating characteristic curve; PPV, positive predictive value; NPV, negative predictive value. LR, Logistic Regression; DT, Decision Tree; RF, Random Forest; XGBoost, eXtreme Gradient Boosting; LightGBM, Light Gradient Boosting Machine; SVM, Support Vector Machine; ANN, Artificial Neural Network.

**Figure 3:**
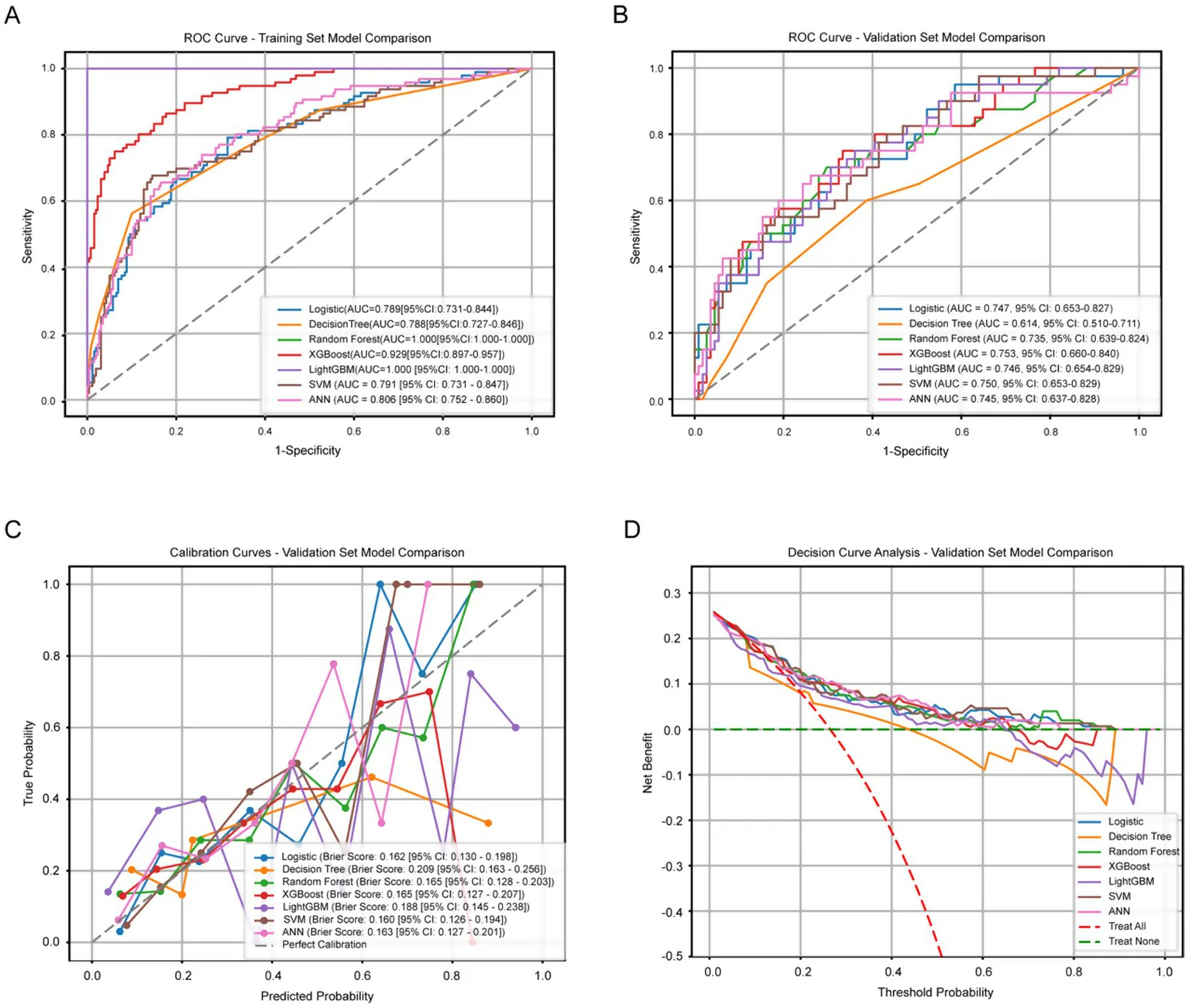
Comprehensive evaluation of machine learning models. **(A)** ROC curves for the seven models in the training cohort. **(B)** ROC curves for the seven models in the validation cohort. **(C)** Calibration curves in the validation cohort, with solid lines representing model-predicted probabilities and the dashed diagonal line representing perfect calibration. **(D)** DCA in the validation cohort, showing the net clinical benefit across a range of threshold probabilities.

### Model interpretation

Figure 4A ranks the importance of each predictor according to the mean absolute SHAP value. Baseline NIHSS score, blood glucose level, age, PLT, NLR, MO were identified as the major contributors to the prediction of a non-excellent functional outcome at 90 days. Among these predictors, baseline NIHSS score showed the highest importance, followed by blood glucose level, age, PLT, NLR, and MO. The SHAP summary plot (Figure 4B) further illustrates the relative importance of each predictor and the direction of its contribution to the model output. Each point represents an individual patient, with color indicating the feature value (low to high) and the SHAP value on the x-axis representing the contribution of the corresponding feature to the model prediction. To further characterize the relationships between individual predictors and their corresponding SHAP values, SHAP dependence plots were generated (Figure 5). Overall, higher values of baseline NIHSS score, blood glucose level, age, platelet count, NLR, and monocyte count were generally associated with more positive SHAP values, indicating that, within the XGBoost model, higher values of these predictors tended to shift the model prediction toward a higher probability of a 90-day non-excellent functional outcome.

**Figure 4:**
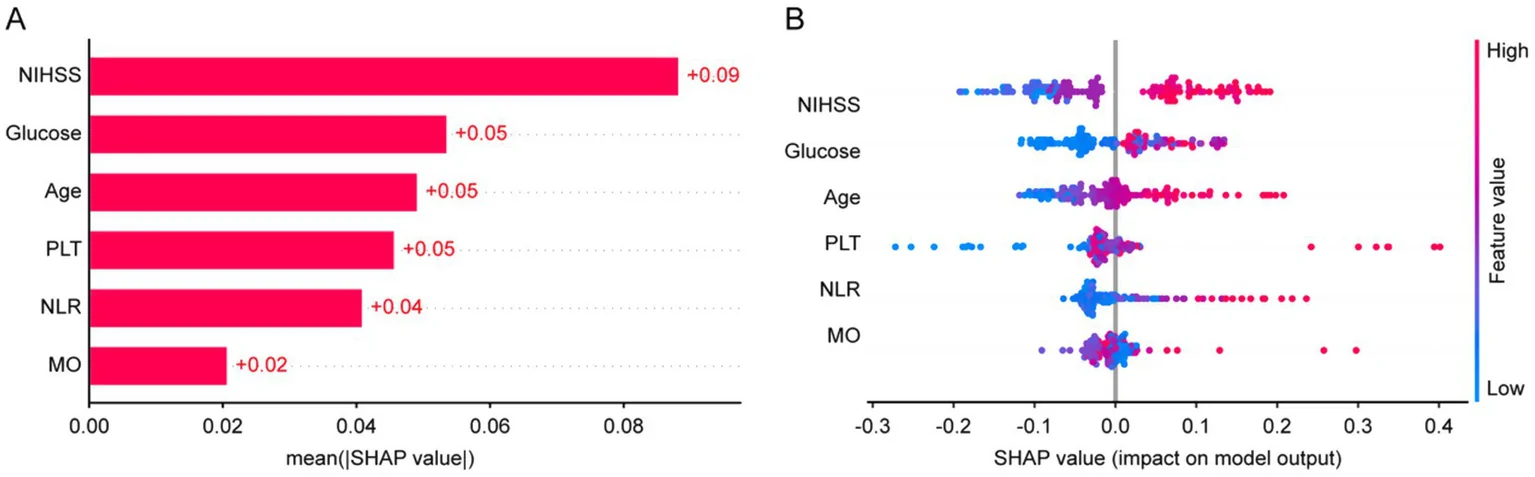
Feature importance analysis by SHAP method for XGBoost model. **(A)** SHAP feature importance ranking based on the mean absolute SHAP value. **(B)** SHAP summary plot of the XGBoost model. NIHSS, National Institutes of Health Stroke Scale; PLT, platelet count; NLR, neutrophil-to-lymphocyte ratio; MO, monocyte count.

**Figure 5:**
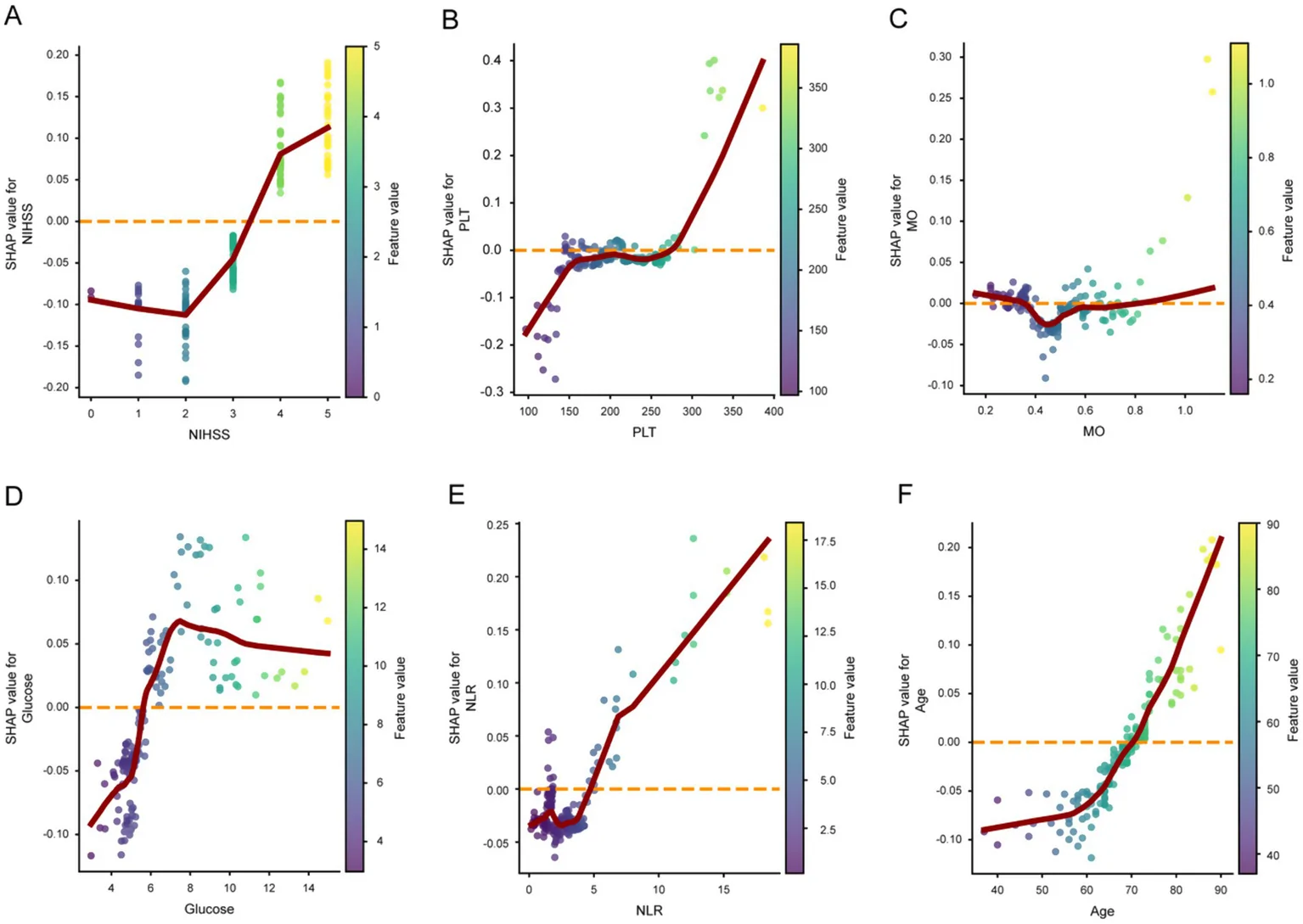
SHAP dependence plots for individual predictors in the XGBoost model. **(A)** NIHSS; **(B)** PLT; **(C)** MO; **(D)** Glucose; **(E)** NLR; **(F)** Age. NIHSS, National Institutes of Health Stroke Scale; PLT, platelet count; NLR, neutrophil-to-lymphocyte ratio; MO, monocyte count.

## Discussion

In this study, we developed and internally validated a machine learning-based prediction model for non-excellent functional outcomes at 90 days in patients with mild acute ischemic stroke undergoing intravenous thrombolysis, using readily available clinical and laboratory variables collected before thrombolysis. The results showed that XGBoost achieved favorable overall predictive performance in terms of discrimination, calibration, and clinical utility, and its interpretability was further enhanced through SHAP analysis. This model enables risk assessment at admission and before thrombolytic therapy, which may facilitate the early identification of patients at higher risk of unfavorable recovery and provide potential support for intensified clinical monitoring, optimized inpatient management, and early rehabilitation assessment. In clinical practice, patients identified as high risk may benefit from closer neurological monitoring to detect early deterioration, more individualized inpatient management to address modifiable risk factors, earlier rehabilitation assessment to facilitate timely recovery planning, and closer follow-up after discharge to monitor functional recovery and treatment needs.

In the present study, baseline NIHSS score was identified as an important predictor of a non-excellent functional outcome at 90 days after intravenous thrombolysis in patients with mild acute ischemic stroke. This finding is consistent with previous studies and further suggests that baseline NIHSS score retains prognostic value even among patients with mild AIS (NIHSS ≤5). Although patients with mild AIS generally present with less severe neurological deficits, a higher baseline NIHSS score may indicate greater initial neurological impairment, increased infarct burden, or more complex underlying pathophysiological conditions, which may be associated with a lower potential for functional recovery (21–23). Furthermore, mild AIS represents a heterogeneous clinical entity, and patients with identical NIHSS scores may have substantially different lesion locations and patterns of functional impairment (24, 25). A slight increase in baseline NIHSS score may reflect deficits involving language, visuospatial function, or higher cortical functions, which may not be fully captured by assessments focusing primarily on motor deficits but can substantially affect long-term independence and quality of life (26). Pre-thrombolytic blood glucose was identified as an important predictor of non-excellent functional outcomes in patients with mild AIS undergoing intravenous thrombolysis. A *post hoc* analysis of the NINDS rt-PA Stroke Trial by Bruno et al. demonstrated that elevated admission blood glucose was independently associated with a lower likelihood of a favorable functional outcome at 90 days and an increased risk of symptomatic intracerebral hemorrhage (27). Subsequent studies have consistently confirmed that higher baseline blood glucose is an important predictor of unfavorable functional outcomes, hemorrhagic transformation, and mortality following intravenous thrombolysis in patients with AIS (28, 29). The underlying mechanisms are likely multifactorial. Acute cerebral ischemia induces a systemic stress response characterized by activation of the sympathetic nervous system and the hypothalamic–pituitary–adrenal axis, leading to increased secretion of catecholamines and cortisol and consequently resulting in stress hyperglycemia (30, 31). In addition, hyperglycemia may exacerbate ischemia–reperfusion injury by promoting oxidative stress, inflammatory responses, lactate accumulation, and disruption of the blood–brain barrier (32–34). Furthermore, elevated blood glucose may impair microvascular reperfusion and increase the risk of symptomatic intracerebral hemorrhage after thrombolysis, thereby attenuating the therapeutic benefits of intravenous thrombolysis (28, 35). Regardless of whether reperfusion therapy is administered, advanced age has consistently been recognized as an independent predictor of unfavorable functional recovery after acute ischemic stroke. Older patients generally exhibit reduced neuroplasticity, diminished collateral circulatory capacity, a greater burden of comorbidities, and an age-related decline in endogenous neurorepair mechanisms following cerebral ischemia, all of which may contribute to poorer neurological recovery (36).

Platelet count has been associated with clinical outcomes after intravenous thrombolysis in patients with acute ischemic stroke. In a subgroup analysis of the China National Stroke Registry II (CNSR II), Yang et al. reported that, within the normal reference range, higher baseline platelet counts were independently associated with an increased risk of unfavorable functional outcomes, stroke recurrence, and all-cause mortality in patients with ischemic stroke or transient ischemic attack (37). The SHAP dependence plot suggested a potential threshold-like pattern, with platelet values above approximately 280–300 × 10^9^/L associated with higher model-predicted risk of non-excellent functional outcomes. This finding should be interpreted as a model-based predictive association rather than evidence of a causal relationship. The biological mechanisms underlying this association may be related to the central role of platelets in thromboinflammation. Following cerebral ischemia, activated platelets rapidly adhere to injured vascular endothelium and interact with leukocytes through adhesion molecules such as P-selectin and glycoprotein Ibα (GPIbα), promoting the formation of platelet–leukocyte aggregates and amplifying the local inflammatory response (38, 39). These interactions contribute to microvascular thrombosis, endothelial dysfunction, and capillary obstruction. Consequently, even when intravenous thrombolysis successfully restores recanalization of the occluded large vessel, microvascular reperfusion may remain impaired, thereby limiting salvage of the ischemic penumbra and ultimately resulting in less favorable neurological recovery (40, 41). Although platelet count itself does not directly reflect platelet activation, an elevated platelet count may indicate a relatively prothrombotic and proinflammatory milieu, which could contribute to poorer functional recovery after ischemic stroke. Several previous studies involving patients treated with intravenous thrombolysis have consistently demonstrated that a higher baseline NLR is associated with an increased risk of symptomatic intracranial hemorrhage and unfavorable functional outcomes at 3 months (42, 43). In the acute phase following stroke, circulating neutrophils are rapidly mobilized to the ischemic brain. Once localized, these cells discharge reactive oxygen species, proteolytic enzymes, matrix metalloproteinase-9 (MMP-9), and neutrophil extracellular traps (NETs) (44, 45). These mediators amplify the local inflammatory response, disrupt the blood–brain barrier (BBB), and promote microvascular thrombosis. Conversely, acute ischemic stroke induces lymphocyte apoptosis and redistribution, resulting in stroke-induced immunosuppression (44, 46). Therefore, an elevated NLR reflects not only excessive activation of the innate immune response but also impaired adaptive immune regulation, indicating a shift toward a pro-inflammatory state that exacerbates secondary brain injury. Even after successful recanalization following intravenous thrombolysis, activated neutrophils and NETs may continue to obstruct the cerebral microvasculature, contributing to the no-reflow phenomenon and limiting effective tissue reperfusion (40). In addition, neutrophil-derived MMP-9 further compromises BBB integrity, thereby substantially increasing the risk of hemorrhagic transformation after thrombolytic therapy (45, 47). Monocytes are key mediators of the innate immune response following cerebral ischemia. Following the onset of stroke, circulating monocytes migrate into the ischemic brain tissue, undergoing differentiation into macrophages that subsequently release diverse pro-inflammatory cytokines. These inflammatory mediators amplify the local inflammatory response, exacerbate secondary brain injury, and ultimately impede neurological recovery (48, 49).

Although substantial progress has been made in developing prognostic models for AIS, several important limitations remain. Bu et al. developed and externally validated an interpretable machine learning model based on 938 patients with AIS treated with intravenous thrombolysis (12). However, because the study included patients with varying stroke severities, the model may not be directly applicable to patients with mild AIS. Abujaber et al. developed a prediction model for 90-day functional outcomes after intravenous thrombolysis that incorporated in-hospital complications, such as urinary tract infection (50). Although the inclusion of these variables improved predictive performance, it limited the model’s utility for early risk stratification because such information is unavailable at the time of admission. In contrast, the present study specifically targeted patients with mild AIS undergoing intravenous thrombolysis and developed a prediction model using routinely available clinical and laboratory variables collected at admission. This design enables early risk stratification and may facilitate timely clinical decision-making, closer monitoring, and individualized patient management. Furthermore, the model was also thoroughly evaluated regarding its discrimination, calibration, and clinical utility. To improve model interpretability, SHAP analysis was applied to characterize the relative contribution and direction of association of each predictor with the model output, thereby providing greater transparency into the model’s prediction process. However, SHAP attributions may be influenced by correlations among predictors. Accordingly, SHAP-derived feature importance should be interpreted as model-specific predictive attribution rather than as an independent causal effect of an individual predictor.

We recognize that this study is subject to certain limitations. First, the retrospective, single-center design may introduce selection bias and limit the generalizability of our findings. Therefore, prospective multicenter studies involving larger and more diverse populations are warranted to assess the applicability of our findings across different clinical settings. Second, model performance was evaluated using an internal validation cohort derived from the same single-center dataset, and no independent external validation was performed. In addition, performance estimates were based on a single stratified 70:30 split and may therefore be sensitive to the specific random partition. Therefore, the generalizability and robustness of the model across different institutions and populations remain uncertain. Future studies should validate the model in independent external cohorts and further assess model stability using repeated resampling procedures. Third, the XGBoost model showed relatively modest sensitivity (0.525) in the validation cohort, indicating that a proportion of patients with non-excellent outcomes were not identified. This may limit its utility as a standalone screening tool for high-risk patients. Therefore, the model should be considered an adjunct to clinical assessment rather than a replacement for clinical judgment. Fourth, the number of patients treated with tenecteplase was limited in our cohort, and the impact of thrombolytic type on functional outcomes could not be reliably evaluated.

## Conclusion

In summary, we constructed and internally validated a machine learning–based predictive model for predicting 90-day non-excellent functional outcomes among patients with mild acute ischemic stroke who received IVT. Across all algorithms tested, the XGBoost model exhibited favorable predictive capability and showed potential as an adjunctive tool for early risk stratification.

## Data Availability

The raw data supporting the conclusions of this article will be made available by the authors, without undue reservation.
