# Supplementary material for: Machine learning-based models to predict 90-day non-excellent functional outcomes among patients with mild acute ischemic stroke after intravenous thrombolysis
Source: Front Neurol. 2026 Oct 19;17:1960403. doi: 10.3389/fneur.2026.1960403 (PMC13724361; doi:10.3389/fneur.2026.1960403)
Supplement: Supplementary file 1 [file Table_1.docx]

Supplementary Table1 The hyperparameter search space and optimal hyperparameters for each algorithm.

| Model | Hyperparameter search space | Optimal hyperparameters |
| --- | --- | --- |
| Decision Tree | max_depth: 3, 5, 10, None; min_samples_split: 5, 10, 20; max_features: sqrt, None; ccp_alpha: 0, 0.01, 0.1 | 'ccp_alpha': 0.01, 'max_depth': None, 'max_features': 'sqrt', 'min_samples_split': 10 |
| Random Forest | n_estimators: 50–450, step 50; max_features: 2, 3 | n_estimators = 300 , max_features = 2 |
| XGBoost | learning_rate: 0.01, 0.1, 0.2; max_depth: 3, 5, 10; n_estimators: 50, 100, 200; subsample: 0.6, 0.8, 1.0 | 'learning_rate': 0.1, 'max_depth': 3, 'n_estimators': 50, 'subsample': 0.8 |
| LightGBM | learning_rate: 0.1, 0.2; num_leaves: 31, 50, 100; n_estimators=100; subsample: 0.6, 0.8, 1.0; colsample_bytree: 0.6, 0.8, 1.0 | 'colsample_bytree': 0.6, 'learning_rate': 0.1, 'n_estimators': 100, 'num_leaves': 31, 'subsample': 0.6 |
| SVM | C=0.1; kernel: linear, rbf; gamma: scale, auto, 0.01; degree: 2, 3, 4 | 'C': 0.1, 'degree': 2, 'gamma': 'scale', 'kernel': 'linear' |
| ANN | hidden_layer_sizes: (25), (50), (100), (10,10), (50,50), (100,100), (50,50,50); activation: relu, tanh, logistic | 'activation': 'logistic', 'hidden_layer_sizes': (100, 100) |

Supplementary Table 2 The predictive performance with 95% confidence intervals of seven models.

| Model | AUC (95% CI) | Accuracy (95% CI) | Sensitivity (95% CI) | Specificity (95% CI) | F1 Score (95% CI) | PPV (95% CI) | NPV (95% CI) |
| --- | --- | --- | --- | --- | --- | --- | --- |
| Logistic | 0.747 (0.653–0.827) | 0.715 (0.649–0.788) | 0.550 (0.391–0.690) | 0.775 (0.698–0.850) | 0.506 (0.370–0.617) | 0.468 (0.325–0.610) | 0.827 (0.758–0.891) |
| Decision Tree | 0.614 (0.510–0.711) | 0.709 (0.636–0.768) | 0.350 (0.204–0.500) | 0.838 (0.765–0.903) | 0.389 (0.229–0.522) | 0.438 (0.258–0.607) | 0.782 (0.708–0.854) |
| Random Forest | 0.735 (0.639–0.824) | 0.722 (0.649–0.795) | 0.525 (0.361–0.676) | 0.793 (0.717–0.863) | 0.500 (0.359–0.615) | 0.477 (0.333–0.625) | 0.822 (0.750–0.893) |
| XGBoost | 0.753 (0.660–0.840) | 0.748 (0.675–0.815) | 0.525 (0.367–0.679) | 0.829 (0.759–0.900) | 0.525 (0.378–0.644) | 0.525 (0.367–0.682) | 0.829 (0.755–0.898) |
| LightGBM | 0.746 (0.654–0.829) | 0.709 (0.636–0.775) | 0.475 (0.308–0.636) | 0.793 (0.720–0.869) | 0.463 (0.312–0.581) | 0.452 (0.290–0.605) | 0.807 (0.734–0.880) |
| SVM | 0.750 (0.653–0.829) | 0.748 (0.682–0.815) | 0.550 (0.386–0.690) | 0.820 (0.748–0.890) | 0.537 (0.395–0.651) | 0.524 (0.371–0.674) | 0.835 (0.764–0.900) |
| ANN | 0.745 (0.637–0.828) | 0.762 (0.695–0.828) | 0.500 (0.343–0.643) | 0.856 (0.786–0.919) | 0.526 (0.370–0.641) | 0.556 (0.389–0.722) | 0.826 (0.758–0.891) |

Supplementary Table 3. Baseline characteristics and standardized mean differences between patients with excellent and non-excellent functional outcomes

|  | Total(n=507) | Excellent outcome(n=371) | Non-excellent outcome(n=136) | p | SMD |
| --- | --- | --- | --- | --- | --- |
| Male (%) | 311 (61.34) | 228 (61.46) | 83 (61.03) | 0.93 | 0.009 |
| HT (%) | 354 (69.82) | 250 (67.39) | 104 (76.47) | 0.062 | 0.203 |
| DM (%) | 159 (31.36) | 107 (28.84) | 52 (38.24) | 0.056 | 0.200 |
| CHD (%) | 225 (44.38) | 157 (42.32) | 68 (50.00) | 0.149 | 0.155 |
| AF (%) | 48 ( 9.47) | 26 ( 7.01) | 22 (16.18) | 0.003 | 0.289 |
| Smoking (%) | 199 (39.25) | 146 (39.35) | 53 (38.97) | 0.938 | 0.008 |
| Alcohol (%) | 133 (26.23) | 97 (26.15) | 36 (26.47) | 0.941 | 0.007 |
| Antiplatelet (%) | 480 (94.67) | 352 (94.88) | 128 (94.12) | 0.909 | 0.033 |
| Responsible artery (%) |  |  |  | 0.627 | 0.095 |
| Anterior circulation | 317 (62.52) | 231 (62.26) | 86 (63.24) |  |  |
| Posterior circulation | 160 (31.56) | 120 (32.35) | 40 (29.41) |  |  |
| AC + PC | 30 ( 5.92) | 20 ( 5.39) | 10 ( 7.35) |  |  |
| TOAST (%) |  |  |  | <0.001 | 0.483 |
| LAA | 259 (51.08) | 182 (49.06) | 77 (56.62) |  |  |
| SAO | 221 (43.59) | 179 (48.25) | 42 (30.88) |  |  |
| CE | 22 ( 4.34) | 7 ( 1.89) | 15 (11.03) |  |  |
| SOE | 5 ( 0.99) | 3 ( 0.81) | 2 ( 1.47) |  |  |
| Age (median [IQR]) | 69.00 [62.00, 74.00] | 68.00 [61.00, 73.00] | 71.00 [66.00, 77.00] | <0.001 | 0.401 |
| OTT (median [IQR]) | 120.00 [72.50, 180.00] | 120.00 [60.00, 155.00] | 120.00 [90.00, 180.00] | 0.067 | 0.211 |
| NIHSS (median [IQR]) | 3.00 [2.00, 4.00] | 3.00 [2.00, 4.00] | 4.00 [3.00, 5.00] | <0.001 | 0.756 |
| SBP (median [IQR]) | 150.00 [136.00, 165.00] | 150.00 [135.00, 164.00] | 155.50 [143.00, 165.00] | 0.003 | 0.263 |
| DBP (median [IQR]) | 80.00 [75.00, 90.00] | 80.00 [74.00, 90.00] | 81.50 [78.75, 90.00] | 0.032 | 0.182 |
| WBC (median [IQR]) | 7.03 [5.70, 8.50] | 6.80 [5.53, 8.16] | 7.52 [6.35, 9.45] | <0.001 | 0.480 |
| PLT (median [IQR]) | 205.00 [169.50, 243.00] | 203.00 [167.50, 241.00] | 209.00 [173.75, 256.25] | 0.05 | 0.275 |
| LYM (median [IQR]) | 1.60 [1.18, 2.14] | 1.61 [1.24, 2.16] | 1.52 [0.99, 2.04] | 0.013 | 0.150 |
| MO (median [IQR]) | 0.46 [0.36, 0.58] | 0.44 [0.35, 0.57] | 0.51 [0.37, 0.64] | 0.001 | 0.38 |
| NE (median [IQR]) | 4.45 [3.33, 5.86] | 4.24 [3.23, 5.54] | 5.13 [3.93, 7.08] | <0.001 | 0.493 |
| ALB (median [IQR]) | 37.80 [36.05, 39.70] | 37.90 [36.40, 39.60] | 37.70 [35.40, 39.90] | 0.35 | 0.149 |
| TC (mean (SD)) | 4.84 (1.18) | 4.85 (1.19) | 4.80 (1.17) | 0.659 | 0.044 |
| TG (median [IQR]) | 1.38 [1.04, 1.89] | 1.39 [1.06, 1.86] | 1.35 [0.93, 2.08] | 0.941 | 0.076 |
| LDL (mean (SD)) | 2.73 (0.90) | 2.74 (0.90) | 2.71 (0.90) | 0.706 | 0.038 |
| HDL (median [IQR]) | 1.08 [0.90, 1.25] | 1.08 [0.90, 1.25] | 1.07 [0.89, 1.25] | 0.653 | 0.041 |
| Glucose (median [IQR]) | 5.61 [4.93, 7.19] | 5.39 [4.82, 6.53] | 6.66 [5.46, 8.52] | <0.001 | 0.460 |
| Hb (median [IQR]) | 130.00 [103.00, 142.00] | 130.00 [100.00, 141.00] | 129.00 [108.75, 143.00] | 0.462 | 0.118 |
| ALT (median [IQR]) | 15.00 [12.00, 21.00] | 16.00 [12.00, 21.00] | 15.00 [11.00, 21.00] | 0.364 | 0.076 |
| AST (median [IQR]) | 18.00 [14.00, 22.00] | 17.00 [14.00, 21.00] | 18.40 [14.00, 22.00] | 0.205 | 0.100 |
| Ca (median [IQR]) | 2.26 [2.19, 2.33] | 2.25 [2.19, 2.32] | 2.26 [2.18, 2.35] | 0.613 | 0.012 |
| K (median [IQR]) | 3.90 [3.60, 4.20] | 3.90 [3.60, 4.15] | 3.90 [3.60, 4.20] | 0.691 | 0.040 |
| NLR (median [IQR]) | 2.69 [1.79, 4.17] | 2.52 [1.71, 3.70] | 3.31 [1.92, 6.24] | <0.001 | 0.540 |
